# P-1516. Impact of a Team-Based Behavioral and Educational Intervention on Influenza and COVID-19 Vaccine Co-Administration During the 2024-2025 Respiratory Virus Season

**DOI:** 10.1093/ofid/ofaf695.1700

**Published:** 2026-01-11

**Authors:** Tiffany A Dickey, Ruth Carrico, Colm Smart, Laura Hendrix, Kathryn Lang, Hagit Kopel, James A Mansi

**Affiliations:** Moderna, Inc., Cambridge, MA; Norton Healthcare, Louisville, Kentucky; VaxCare LLC, Hooksett, New Hampshire; Moderna, Inc., Cambridge, MA; VaxCare, San Marcos, CA; Moderna, Inc., Cambridge, MA; Moderna, Inc., Cambridge, MA

## Abstract

**Background:**

Despite national recommendations, adult respiratory vaccination rates in the U.S. remain suboptimal, contributing to preventable morbidity and mortality. Co-administering influenza and COVID-19 vaccines is a practical, yet underutilized strategy to improve uptake.

**Methods:**

This project assessed the impact of a team-based behavioral and educational intervention program on influenza and COVID-19 vaccine co-administration. Twenty U.S.-based outpatient clinics serving diverse adult populations were selected for the intervention group and matched to control clinics by vaccination history and demographics. All clinics operated within a technology-enabled vaccine administration ecosystem. Clinic staff (healthcare providers [HCPs] and non-HCPs) at intervention sites participated in the program, consisting of expert-led educational and behavioral interventions. Their perceptions and recommendation behaviors were assessed pre- and post-intervention using a modified Vaccine Confidence Index survey. Adult vaccination data were collected from August 2024 to February 2025. Co-administration rates (receipt of both vaccines within 24 hours) were compared between groups. Chi-square testing and relative risk (RR) estimates with 95% confidence intervals (CI) were calculated.

**Results:**

23,430 and 91,924 adults (≥18 years) received an influenza vaccine in the intervention and control groups, respectively. Of these, 31.4% in intervention clinics and 21.4% in control clinics received a COVID-19 vaccine at the same visit (RR, 1.47; 95% CI, 1.43–1.50; p< 0.001). Among adults ≥65 years, 11,694 and 47,816 received an influenza vaccine in the intervention and control groups, respectively; co-administration rates were 32.1% versus 22.8% (RR, 1.41; 95% CI, 1.37–1.45; p< 0.001). Survey findings showed increased vaccine confidence and recommendation behaviors among intervention clinic staff.

**Conclusion:**

A targeted seasonal program for clinic teams can significantly improve co-administration. These findings underscore the importance of staff behavioral and educational interventions. Further, it highlights their role in reducing missed opportunities for COVID-19 vaccination and strengthening adult immunization efforts.

**Disclosures:**

Tiffany A. Dickey, PharmD, BCIDP, Moderna, Inc.: Employee|Moderna, Inc.: Stocks/Bonds (Public Company) Ruth Carrico, PhD, DNP, APRN, VaxCare: Advisor/Consultant Colm Smart, MBA, VaXcare: Stocks/Bonds (Private Company) Laura Hendrix, MS, Moderna, Inc.: Employee|Moderna, Inc.: Stocks/Bonds (Public Company) Kathryn Lang, MD, PhD, VaxCare: Advisor/Consultant Hagit Kopel, PhD, Moderna, Inc.: Employee|Moderna, Inc.: Stocks/Bonds (Public Company) James A. Mansi, PhD, Moderna, Inc.: Employee|Moderna, Inc.: Stocks/Bonds (Public Company)

